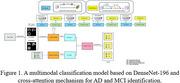# Deep Learning‐Based Identification Model for Alzheimer's Disease and Mild Cognitive Impairment Using Multimodal Information

**DOI:** 10.1002/alz70860_100850

**Published:** 2025-12-23

**Authors:** Nini Rao, Boyang Yu

**Affiliations:** ^1^ University of Electronic Science and Technology of China, CHengdu, Sichuan, China

## Abstract

**Background:**

Alzheimer's disease (AD) is a prevalent neurodegenerative disorder, and its early diagnosis is critical for effective intervention and patient management. This study aims to integrate MRI imaging data and clinical information to develop a deep learning‐based classification model for Alzheimer's disease and mild cognitive impairment (MCI), providing auxiliary support for clinical diagnosis.

**Method:**

Structural images of brain gray matter and white matter were first extracted from patients' MRI scans. The images and clinical data underwent preprocessing steps, including standardization, data augmentation, and region of interest (ROI) extraction. A multimodal classification model based on DenseNet‐196 and a cross‐attention mechanism was then developed for AD and MCI identification, as shown in Figure 1. The proposed method was evaluated using the publicly available ADNI (Alzheimer's Disease Neuroimaging Initiative) dataset.

**Result:**

The 5‐fold cross‐validation experiments demonstrated robust performance, with the proposed model achieving an average accuracy of 96.39%, sensitivity of 94.44%, specificity of 98.28%, F1 score of 0.9577, and AUC of 0.9728 on the validation set. The Grad‐CAM (Gradient‐weighted Class Activation Mapping) visualization revealed that the model primarily focused on the hippocampus and frontal lobe regions when identifying AD, which are closely associated with the disease's pathological changes.

**Conclusion:**

The proposed model effectively extracts the key features of Alzheimer's disease, offering a promising approach for early diagnosis and precision treatment. Additionally, it aids clinicians in understanding the pathogenesis of AD and MCI, providing valuable insights for disease management.